# Genetic Diversity of the Amur Tiger (*Panthera tigris altaica*): Are There Differences between the 20th and the Early 21st Centuries?

**DOI:** 10.1134/S001249662370059X

**Published:** 2023-12-12

**Authors:** P. A. Sorokin, V. G. Yudin, S. V. Naidenko, J. A. Hernandez-Blanco, M. D. Chistopolova, V. V. Rozhnov

**Affiliations:** 1 Severtsov Institute of Ecology and Evolution, Russian Academy of Sciences, Moscow, Russia; 2 Federal Scientific Center of the East Asian Terrestrial Biodiversity, Far Eastern Branch, Russian Academy of Sciences, Vladivostok, Russia

**Keywords:** Sikhote-Alin population, historical and modern samples, bottleneck

## Abstract

Polymorphism of nine microsatellite loci in the Sikhote-Alin tiger population was analyzed in the modern and recent historical periods, using blood, excrement, and museum bone samples. Tests for excess heterozygosity to determine whether the population went through a period of low abundance and a low value of the Garza–Williamson coefficient indicated that such events were highly probable to occur in both recent and earlier history. The mean effective population size *Ne* of a current sample was 34.4 (95% confidence interval 17–106.8). This fact is of great interest in the contest of conservation and restoration of endangered large cat species.

## INTRODUCTION

**Fig. 1.**
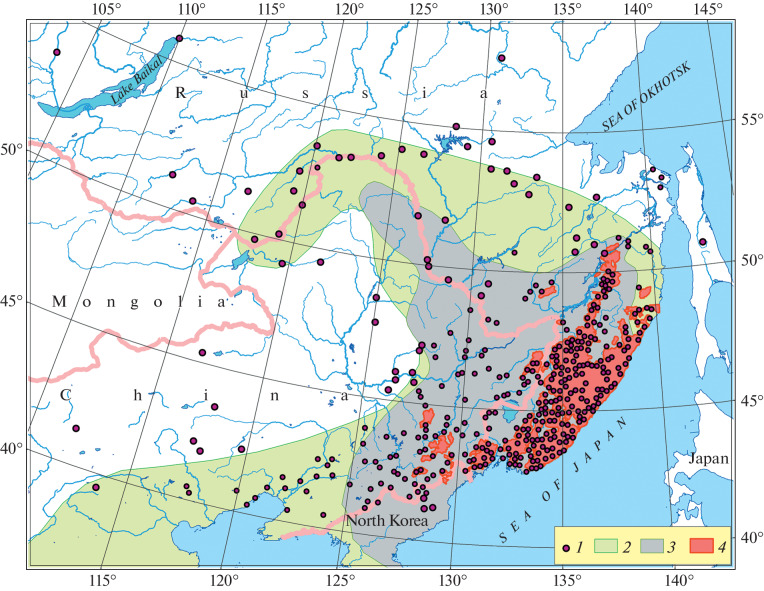
Amur tiger distribution [1]: *1*, sites where Amur tigers were spotted historically (according to the literature); *2*, historical Amur tiger region; *3*, species region at the end of the 19th century; *4*, modern Amur tiger region.

Substantial changed occurred in the Amur tiger (*Panthera tigris altaica*) Temminck, 1844)) population and its range in the 20th century. Data on Amur tiger recordings from various sources have been summarized to map the range of the subspecies and to analyze its dynamics over a 100-year period [1]. Historically, the tiger species range was rather broad (Fig. 1) and included Northeastern China, Primorsky krai, southern Khabarovsk krai, Jewish Autonomous Region, and Amurskaya Region; single tigers reached Transbaikalia. The range has been greatly reduced and become discontinuous by now.

Dramatic changes in Amur tiger population size accompanied the changes in the tiger range. There is established opinion that the tiger population size reached its minimum (30–40 tigers) as a result of killing in the 1940s [2] and then substantially increased over a short period of time to 500 tigers by 1990 [3] and 523–540 tigers by 2015 [4].

To what extent did the past events affect the genetic variation in Amur tiger? To answer the question, we compared the genetic variation of Amur tiger in historical retrospective, between the 20th and early 21st centuries.

## MATERIALS AND METHODS

To perform molecular genetic analyses, DNA was isolated from Amur tiger sculls stored in the collections of the Zoological Museum (Moscow State University) and Zoological Institute (Russian Academy of Sciences) and fecal, hair, and blood samples, which we collected as part of the “Program of Amur Tiger Research in the Russian Far East,” within the framework of a permanent expedition of the Russian Academy of Sciences to investigate the animals included in the Red Book of the Russian Federation and other especially important animals of Russian fauna.

A historical sample included 61 specimens from Amur tiger sculls collected in Primorsky and Khabarovsk krais from 1938 to 1994 (Table 1). Molecular genetic data obtained with museum specimens of only 22 Amur tigers that had more comprehensive genetic profiles were used in further comparisons with the data that have been obtained for 44 tigers of a modern sample (from 2009 to 2013) and published previously [5]. Genetic variation of the subspecies in the period from 1938 to 1994 is reflected in the results of molecular genetic analyses of the historical specimens.

**Table 1.** Historical museum specimens of Amur tiger that were used in molecular genetic analyses

Specimen	Killing date	Killing and storage site
36 332	Dec. 19, 1981	Primorsky krai, Spassk raion, village Novovladimirovka
36 333	1978	Primorsky krai, Ternei raion
36 335	February 1980	Primorsky krai, Olga raion
36 336	Jan. 14, 1987	Primorsky krai, Khasanskii raion
36 342	Oct. 12, 1981	Primorsky krai, Shkotovo raion
36 345	Nov. 27, 1991	Primorsky krai, Partizansk raion, village Novoe
36 347	1993–1994	Primorsky krai, Lesozavodsk raion
36 351	February 1984	Primorsky krai, Pozharskii raion, village Krasnyi Yar
36 353	Nov. 10, 1981	Primorsky krai, Olga raion
36 354	1983–1984	Primorsky krai, Chuguevka raion
36 358	1965	Primorsky krai, Imanskii raion
36 361	Jan. 12, 1987	Primorsky krai, Shkotovo raion, village Smolyaninovo
36 367	Nov. 17, 1982	Primorsky krai, Chernigovka raion, village Gornyi Khutor
36 371	Jun. 21, 1986	Primorsky krai, Ussuriisk Nature Reserve
36 375	April 1984	Primorsky krai, Anuchino raion
36 376	January 1988	Primorsky krai, Chuguevka raion
36 379	Apr. 7, 1984	Primorsky krai, Partizansk raion
36 380	Dec. 28, 1986	Primorsky krai, near Vladivostok
s34 855	1938	Primorsky krai, Krasnoarmeisk raion, village Novopokrovka, collection of Zoological Museum
s91 581	1966	Primorsky krai, Nakhodka, collection of Zoological Museum
s96 811	1972	Primorsky krai, Lazo raion, collection of Zoological Museum
s100 000	February 1974	Primorsky krai, Dal’nerechensk raion, collection of Zoological Museum

Specimens 36332–36380 from the collection of the Zoological Institute were collected by V.G. Yudin.

**Fig. 2.**
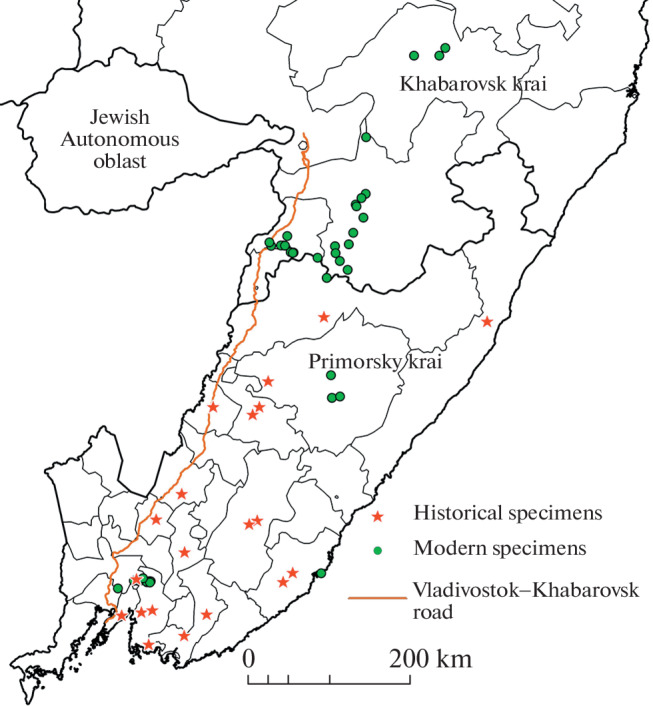
Collection sites of sculls from museum collections and Amur tiger specimens for genetic analysis.

The same microsatellite loci were examined in the historical and modern samples; sample localities were restricted to the region of the Sikhote-Alin subpopulation (Fig. 2).

Bone tissue samples were taken from the mandible with a Proxxon instrument (Germany) and a metal drill of 1–1.2 mm in diameter. Ground bone tissue samples were stored at –20°C. DNA was isolated using a QIAamp DNA investigator kit (Qiagen, Germany) according to a bone DNA isolation protocol.

Nuclear DNA was tested as described previously [5]. PCR with all primers was performed in four replicas. A generalized genotype was established when a heterozygous allele was repeated two times and a homozygous allele, three times. Changes in allele frequencies were evaluated using Fisher’s test and considered significant at *p* < 0.05. To find out if a bottleneck occurred in the population, tests for excess heterozygosity were carried out using Bottleneck, v. 1.2.0.2 and the IAM, TPM, and SMM mutation models [6]. The expected heterozygosity *He*, observed heterozygosity *Ho*, and M-statistic [7] were calculated using Arlequin v. 3.5.1.2 [8]. The effective population size *Ne* was calculated by the linkage disequilibrium (LD) method, using NeEstimator v. 2 [9]. The allelic richness was calculated using Fstat 294 [10].

## RESULTS AND DISCUSSION

In total, genetic profiles were obtained for 22 museum and 44 modern specimens of Amur tigers.

The Amur tiger population was characterized with respect to allele frequencies of nine microsatellite loci (Table 2), effective number of alleles *Ar*, expected heterozygosity *He*, observed heterozygosity *Ho*, and Garza–Williamson coefficient *M* (Table 3).

**Table 2.** Allele frequencies (%) of nine microsatellite loci in the Sikhote-Alin subpopulation

Locus	Allele	Modern sample	Historical sample
e7	150	6.10	6.82
	152	90.24	90.91
	156	3.66	2.27
fca304	128	33.33	20.45
	134	34.52	45.45
	136	32.14	34.09
fca43	117	0	4.55
	119	11.36	18.18
	123*	59.09	38.64
	127	29.55	38.64
e21b	160	63.64	59.52
	162	13.94	21.43
	164	22.73	19.05
pun935	102	64	59.09
	108	34.67	31.82
	120	1.33	2.27
	124	0	6.82
fca5	139*	38.64	61.36
	141	36.36	25.00
	143*	25.00	9.09
	145	0	4.55
fca161	184	4.65	15.00
	188	1.16	0
	190*	70.93	45.00
	192*	23.26	40.00
fca91	134	2.50	0
	140	73.75	86.84
	144	23.75	13.16
fca441	144	15.85	15.91
	148	30.49	36.36
	152	32.93	25.00
	156	3.66	0
	160	15.85	18.18
	164	1.22	4.55

* Alleles that significantly differ in frequency between the samples.

**Table 3.** Allelic diversity *Ar*, expected heterozygosity *He*, observed heterozygosity *Ho*, and Garza–Williamson coefficient *M* in the historical (hist.) and modern (mod.) samples

Locus	*Ar* (hist.)	*Ar* (mod.)	*Ho* (hist.)	*He* (hist.)	*Ho* (mod.)	*He* (mod.)	*M* (hist.)	*M* (mod.)
e7	2.862	2.999	0.09091	0.17230	0.19512	0.18278	0.42857	0.42857
fca304	3.000	3.000	0.36364	0.65011	0.69048	0.67441	0.33333	0.33333
fca43	3.984	3.000	0.50000	0.68182	0.59091	0.55695	0.36364	0.33333
e21b	3.000	3.000	0.57143	0.57724	0.61364	0.53083	0.60000	0.60000
pun935	3.862	3.000	0.63636	0.55708	0.58333	0.47640	0.17391	0.15789
fca5	3.984	3.000	0.36364	0.56342	0.72727	0.66353	0.57143	0.60000
fca161	3.000	3.837	0.45000	0.63077	0.44186	0.44569	0.33333	0.44444
fca91	2.000	2.991	0.26316	0.23471	0.37500	0.40411	0.40000	0.27273
fca441	4.984	5.877	0.72727	0.76216	0.95122	0.75610	0.23810	0.28571
Mean			0.44071	0.53662	0.57431	0.52120	0.38248	0.38400
s.d.			0.19585	0.20011	0.21849	0.17184	0.13919	0.14885

A decrease allelic diversity (*Ar*) with a simultaneous dramatic decrease in population size reduces rare alleles [11]. Our analysis of the two samples showed an increase in *Ar* by one allele for three loci and a decrease for three loci (Table 3). The frequency of such an allele was lower than 7% in all cases. Opposite changes allelic diversity values indicate that our samples of specimens most likely failed to fully represent genetic diversity of the population in different periods of time. This is because migration, but not an accumulation of mutations in the genome, is the only factor that can explain an increase in allele number in the modern population compared with the historical population. A significant change in allele frequency between the two samples was observed for five alleles of three loci.

The effective population size *Ne* was calculated to be 11.4 (95% interval 5.1–28.8) for the historical sample and 34.4 (95% interval 17–106.8) for the modern sample. A comparable *Ne* value, 26 tigers (95% interval 12–79), has been calculated for a sample of 2001–2002 by the LD method [12]. A far lower effective population size obtained for the historical sample possibly indicates that the population size was substantially lower than modern in the respective period.

A check for excess of heterozygosity was performed with the modern sample by the Wilcoxon test to determine whether a bottleneck occurred in the population. Significant results were obtained with all of the mutation models: IAM (*P* = 0.00098), TPM (*P* = 0.00098), and SMM (*P* = 0.00098). Similar results were obtained with the historical sample: IAM (*P* = 0.00098), TPM (*P* = 0.00098), and SMM (*P* = 0.00195). Other tests confirmed a recent bottleneck. Likewise, a significant excess of heterozygosity by the Wilcoxon test (*P* < 0.001) has been observed for a modern sample of 15 tigers (1999–2000) [13].

The Garza–Williamson tests estimates the likelihood of a bottleneck for an earlier historical period (more than 100 generations ago). The coefficient *M* of both of the samples was 0.38, substantially lower than the threshold *M* = 0.68, which is characteristic of presumably stable populations [7]. A bottleneck occurring in that period has not been supported in other studies, where the coefficient *M* has been estimated at 0.67 [12] or 0.835 [13]. Three haplotypes have been observed in the modern population in a mitochondrial DNA analysis, the result supporting a bottleneck [14]. In addition, this might be associated with an initially small size of the founder population that migrated from Central Asia to the modern Amur tiger range approximately 10 000 years ago [15].

Thus, in contrast to other studies, two basically different models confirmed in our work that a low-abundance period occurred in the history of the population. The population was presumably bottlenecked twice, in the remote past (approximately 10 000 years ago [14]) and recently (in the mid-20th century [2]).
